# Structure-function analysis of Cdc25^Twine^ degradation at the *Drosophila* maternal-to-zygotic transition

**DOI:** 10.1080/19336934.2022.2043095

**Published:** 2022-02-28

**Authors:** Patrick L. Ferree, Maggie Xing, Jenny Q. Zhang, Stefano Di Talia

**Affiliations:** a Department of Cell Biology, Duke University Medical Center, Durham, NC, USA; b Department of Surgery, Alpert Medical School, Brown University, Providence, RI, USA

**Keywords:** Embryonic development, Cdc25, protein degradation, cell cycle

## Abstract

Downregulation of protein phosphatase Cdc25^Twine^ activity is linked to remodelling of the cell cycle during the *Drosophila* maternal-to-zygotic transition (MZT). Here, we present a structure-function analysis of Cdc25^Twine^. We use chimeras to show that the N-terminus regions of Cdc25^Twine^ and Cdc25^String^ control their differential degradation dynamics. Deletion of different regions of Cdc25^Twine^ reveals a putative domain involved in and required for its rapid degradation during the MZT. Notably, a very similar domain is present in Cdc25^String^ and deletion of the DNA replication checkpoint results in similar dynamics of degradation of both Cdc25^String^ and Cdc25^Twine^. Finally, we show that Cdc25^Twine^ degradation is delayed in embryos lacking the left arm of chromosome III. Thus, we propose a model for the differential regulation of Cdc25 at the *Drosophila* MZT.

## Introduction

The earliest stage of embryonic development in most metazoans involves a series of extremely fast cell cycles punctuated by the onset of morphogenesis [1]. For instance, the nucleus of the fertilized *Drosophila* egg reproduces itself, over thirteen nuclear cycles, into about six thousand nuclei in just two hours, at which point there is a major transition in the cell cycle. Whereas the initial thirteen nuclear cycles are meta-synchronous, syncytial, and largely abbreviated (composed only of S- and M-phases), the fourteenth round of cell divisions follow cellularization, a specialized form of cytokinesis, and include an extended gap phase (G2) following DNA synthesis [2].

Central to our current understanding of this cell-cycle transition is the much more global transition from maternal to zygotic (MZT) control of embryogenesis. At fertilization, many of the important molecules needed for driving the thirteen nuclear cycles have been synthesized and provided by the mother, including, for instance, mRNAs, proteins, and a significant portion of nucleotides [2–4]. But during the MZT many of these maternally deposited mRNAs and proteins are targeted for degradation and the expression of zygotic genes increases dramatically.

It was hypothesized early on that a candidate mechanism that could explain the lengthening of the cell cycle during the *Drosophila* MZT might involve zygotic downregulation of certain mitotic activators. Support for this idea came with the discovery that the number of early nuclear cycles in the fly embryo is linked to the appropriately timed downregulation of mRNAs that code for Cdc25 tyrosine phosphatases [5]. Cdc25 proteins are involved in a well described molecular circuit that regulates mitotic activity [6]. At the centre of this circuit is the master regulator of mitosis called cyclin dependent kinase 1 (Cdk1), which, when bound to a cyclin partner, may be activated or inactivated like a switch based on its phosphorylation state [6]. Whereas Wee1 and Myt1 kinases place inhibitory phosphates at Cdk1ʹs ATP-binding site, Cdc25 proteins exactly counter this operation by removing the phosphates [7,8]. By this logic the downregulation of Cdc25 activity during the MZT allows for the accumulation of inhibitory phosphates on the cyclin-Cdk1 complexes [6], which prevents further nuclear cycles and transitions cells instead into a G2 phase. Thus, the mechanisms that control the activity of Cdc25 are central in the timing of the cell-cycle transition at the MZT.

**Figure 1.**
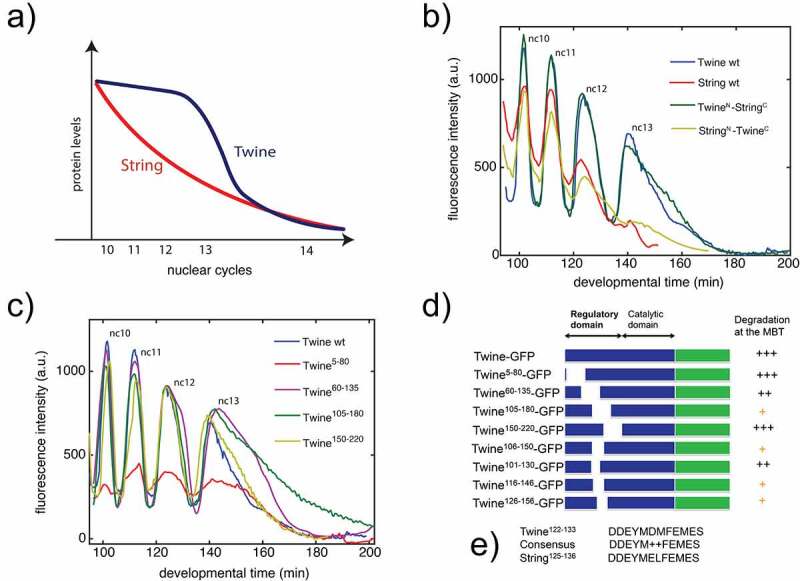
Domain analysis of Cdc25^Twine^. (a) Schematic illustration of distinct protein dynamics of Cdc25^Twine^ and Cdc25^String^ prior to the MZT. (b) Quantification of dynamics of Cdc25^Twine^-GFP, Cdc25^String^-GFP, and two chimeras. (c) Quantification of dynamics of Cdc25^Twine^-GFP constructs that are missing various sections of the N-terminus. (d) Cdc25^Twine^-GFP constructs and their ability to degrade prior to the MZT. (e) Consensus sequence in Cdc25^Twine^ and Cdc25^String^.

There are two Cdc25 proteins in *Drosophila*: Cdc25^String^ and Cdc25^Twine^ [5]. Although both are expressed in the oocyte, Cdc25^Twine^ alone is essential for driving the early nuclear cycles [5], as well as instances of meiosis, but is not required for cell proliferation in later stages of development [9,10]. Both homologs are present as mRNA and protein in the early embryo and their dynamics have been characterized in detail. One important observation is that whereas Cdc25^String^ proteins are gradually degraded leading into the MZT, the degradation pattern of Cdc25^Twine^ proteins is much more switch-like, which we have illustrated schematically in Figure 1a. Several experiments led to the hypothesis that – contrary to the earlier model based on the downregulation in the concentration of *cdc25* mRNA – the termination of the early nuclear cycles may be linked instead to the abrupt degradation of Cdc25^Twine^ proteins [11,12]. Further evidence that supports this idea is that expression of a Cdc25^Twine^ mutant with delayed degradation kinetics results in premature and unscheduled nuclear divisions in a large fraction of embryos [11]. Stabilized mRNA can also cause premature divisions prior to completion of cellularization and gastrulation [13]. The central role of protein degradation is also supported by the observation that *cdc25^twine^* mRNA outlives the switch-like degradation of Cdc25^Twine^ proteins [11,12]. While these data argue for a role of protein degradation, post-translational modifications that downregulate the enzymatic activity of Cdc25^Twine^ could also be important and in fact degradation might be a byproduct of such regulation. For instance, modifications from the protein phosphatase *PpV* have been recently implicated in the regulation of the initial levels of Cdc25^Twine^ present in the embryo [14]. However, these modifications do not seem to contribute to the dynamics of degradation, but rather the levels of protein loaded in the embryo maternally. In this paper, we specifically focus on the timing of degradation. The molecules involved in targeting Cdc25^Twine^ for degradation are not fully known. It was shown that both the remodelling of the cell cycle at the *Drosophila* MZT and the abrupt degradation of Cdc25^Twine^ are linked to onset of zygotic gene transcription, particularly, the transcription of the pseudo-kinase *tribbles* [12]. The primary purpose of this paper is to identify regions of the Cdc25^Twine^ protein that mediate its timely degradation.

## Materials and methods

### Reagents

Standard methods were used throughout. Here is a comprehensive list of the alleles and stocks that were used along with their Fly Base [15] identification number: *UASt-stgGFP* (FBal026977), *UASt-tweGFP* (FBal0286379), *UASt-stgDronpa* (FBal0270090), *UASt-tweDronpa* (FBal0286381), *His2Av-RFP* (FBal0247236), *matα4-GAL4-VP16* (FBal0086843), *grp^209^* (FBal0197284), *grb^z5170^* [16], *lok^30^* (FBal0197286), and the deficiency line Df(3 L)BSC839 (FBst0027917). Both the compound chromosome stock RM(3 L); RM(3 R) (=C(3)se) and the *TM3.hb-GFP* balancer stock were gifts from the Wieschaus lab at Princeton. Plasmids *UASt-stg-tweGFP* and *UASt-twe-stgGFP* (Cdc25^String^/Cdc25^Twine^ chimera constructs) were generated using PCR-splice overlap. Plasmids of the form *UASt-twe^deletion^-GFP* were generated using PCR-splice overlap. The *tweGFP* lines were generated using a genomic transgene inserted in attP40 (25C6) or attP2 (68A4) landing site. The *tweGFP* construct itself was synthesized by Genscript, and consists of a 3.7 kb *twe* rescue fragment [9] as well as a C-terminal fusion of *twe* with *EGFP*. This construct was then inserted into the vector pBabr by standard restriction digest and ligation, and the resulting *pBabr-tweGFP* constructs were injected into flies by Genscript.

### Genetic crosses

Embryos maternally expressing fluorescently tagged Cdc25^Twine^ and Cdc25^String^ type constructs (including chimeras and deletions) were obtained from the F1 generation of a cross between females carrying the UASt transgenes and males carrying Gal4 and His2Av-RFP (+/Y; *His2Av-RFP matα4-GAL4-VP16/His2Av-RFP matα4-GAL4-VP16; matα4-GAL4-VP16/matα4-GAL4-VP16*). DNA-checkpoint mutant embryos were trans-heterozygous for *grapes* (*grp^z5170^/grp^209^)* and homozygous for *loki* (*lok^30^*) [16]. Those embryos were obtained from females with the following genotype: +/+; *grp^z5170^lok^30^/grp^209^lok^30^; UASt-tweGFP(Dronpa) (UASt-stgGFP(Dronpa))/His2Av-RFP matα4-GAL4-VP16*. Such females were generated by crossing +/+; *grp^z5170^lok^30/^CyO; UASt-tweGFP (UASt-stgGFP)/TM3* females with *+/Y; grp^209^lok^30^/CyO; His2Av-RFP matα4-GAL4-VP16/TM3* males. Embryos lacking the left arm of chromosome III (3 L^−^ embryos) were obtained from a compound chromosome III stock RM(3 L); RM(3 R) (=C(3)se) also carrying the *tweGFP* rescue construct on the second chromosome. Embryos homozygous for a deficiency Df(3 L)BSC839 covering the *tribbles* locus (maternally expressing fluorescently tagged Cdc25^Twine^) were obtained from the following genotype: +/+; *tweGFP, His2Av-RFP/CyO; Df(3 L)BSC839*/*TM3, hb-GFP*, where the TM3 balancer is marked with hb-GFP. With this experimental set up, one quarter of the embryos were expected to be homozygous for the deficiency and they could be identified by the lack of the hb-GFP expression in the anterior half of the embryo by late cell cycle 14, when tweGFP has been fully degraded.

### Microscopy

**Figure 2.**
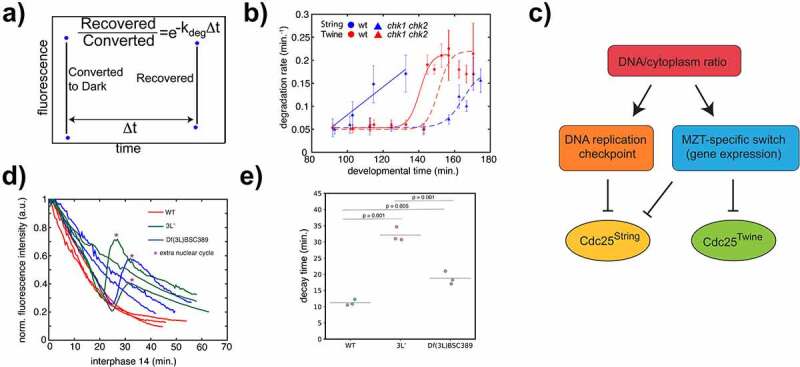
Model for differential regulation of Cdc25^Twine^ and Cdc25^String^. (a) Conceptual illustration of how protein degradation rate is measured [11]. (b) Degradation rates as a function of developmental time for Cdc25^Twine^ and Cdc25^String^ in wild-type and DNA-checkpoint mutant backgrounds. (c) Proposed model for differential regulation of Cdc25^Twine^ and Cdc25^String^ at the MZT. (d) Quantification of Cdc25^Twine^-GFP dynamics in wild-type, flies having only the left arm of their chromosome III, and flies homozygous for a deficiency that covers the *tribbles* locus. (e) Cdc25^Twine^-GFP decay times for each of the samples in (D) were calculated by fitting them with an exponential function and solving for the lifetime (Supp. Figure 1). Statistical significance was determined using a one-way ANOVA followed by a Tukey’s test to compare the pairs.

Quantitative live imaging experiments were performed using either an SP5 (Figure 1b and c) or SP8 (Figure 2d) confocal microscope from LEICA. In the case of the SP5, the following specifications apply: a 20X/0.7 numerical aperture glycol-immersion objective, an argon ion laser, and a 405-nm and a 594-nm diode lasers. In the case of the SP8, the following specifications apply: a 20X/0.75 numerical aperture oil-immersion objective, an argon ion laser, and a 561-nm diode laser. For embryos expressing the fluorescently tagged Cdc25^Twine^ and Cdc25^String^ type constructs, images (1024x512 pixels) were acquired at a frame rate of about 1/20 seconds. A variable number of images (depending on the level of signal) were averaged together to reach a satisfactory signal-to-noise ratio. The 405-nm laser of the SP5 was used to covert the Dronpa from its dark to fluorescent state. The conversion of the Dronpa from fluorescence to dark was performed using the 488-nm and 496-nm light from the argon ion laser of the SP5. Dronpa fluorescent images were acquired with the 496-nm excitation light along with sufficient averaging over images.

### Data and image analysis

Custom MATLAB software was used to segment nuclei based on His2Av-RFP signal. Segmentation was used to generate a mask that was then used to estimate the nuclear fluorescence intensity of the tagged constructs.

### Measurements of Cdc25^String^-Dronpa and Cdc25^Twine^-Dronpa

Dronpa is a photoswitchable fluorescent molecule that when irradiated with a 488-nm laser enters a dark state, but is restored to a light state when irradiated with 405-nm laser [17]. In living tissue, one can convert the Dronpa molecules (generally fused to proteins of interest) in a particular region to the dark state, and then at a later time restore the light state. In doing so, one may calculate the converted/restored ratio, and determine a protein’s effective rate of decay. Here, we use Dronpa to estimate the degradation rate of certain cell cycle regulators in the early nuclear cycles. Because the nuclear envelope breaks down during each nuclear cycle, the nuclear levels of Cdc25^Twine^ and Cdc25^String^ are very dynamic and complicate the measurement of the converted/recovered ratio. However, the peak in nuclear concentration during each cycle is stable for about 1–2 minutes and is consistently achieved about 4 minutes after Nuclear Envelope Formation (NEF) [11]. Therefore, we determine protein’s degradation rate per nuclear cycle, by converting to the dark state with illumination with the 488-nm laser at the peak of one cycle (4 minutes after NEF), and then restoring to the light state at the peak of the next cycle using the 405-nm laser (4 minutes after NEF).

## Results and discussion

We first set out to identify which general region of Cdc25^Twine^ is responsible for targeting it for degradation. A general feature of Cdc25 phosphatases, shared by Cdc25^String^ and Cdc25^Twine^, is that they contain a well-conserved catalytic domain in the C-terminus and a divergent regulatory domain in the N-terminus [18]. Therefore, we suspected that the differential dynamics of Cdc25^Twine^ and Cdc25^String^ are most likely encoded by the N-terminus of the proteins. To test this hypothesis, we measured the dynamics of GFP tagged chimeras built from the N-terminus of Cdc25^Twine^ and C-terminus of Cdc25^String^ and vice versa (Figure 1b). The oscillatory nature of the dynamics reflects the fact that the reporters are dispersed at nuclear envelope breakdown between each nuclear cycle (Figure 1b). But the peak of fluorescence intensity during each cell cycle can be taken to represent the concentration of the reporter. As expected, we found that the dynamics were controlled by the N-terminus regions (Figure 1b).

Next, we set out to narrow down the region in the N-terminus responsible for targeting Cdc25^Twine^ for degradation. We built a series of Cdc25^Twine^-GFP fusion proteins with various sections of the N-terminus removed (Figure 1d). We measured the nuclear levels over time for each fusion protein during the early embryonic cell cycles (Figure 1c). The nuclear levels of the Twine^5−80^ construct are likely decreased because the Cdc25^Twine^ protein has two NLS sequences, one of which is located at 36–41. However, its degradation dynamics is similar to the wild type protein. We found that the timing of the degradation was significantly delayed in two of the constructs: Twine^60−135^ and Twine^105−180^ (Figure 1c). Based on these observations and on similar ones using smaller deletions, we narrowed down the region of the N-terminus to about 20–30 amino acids.

While we were unable to identify any known domain or putative post-translational modifications in the region responsible for degradation – including, notably, the phosphorylation sites recently identified that control Cdc25^Twine^ levels [14] – we did identify a 12 amino acid sequence that is highly conserved in both Cdc25^String^ and Cdc25^Twine^ (Figure 1e). This led us to speculate that perhaps both Cdc25s would be targeted for degradation similarly at the MZT and that such degradation is not observed in Cdc25^String^ because it is normally degraded prior to the MZT. To test this hypothesis, we used a photoinducible Dronpa system [17,19,20] (Figure 2a) to measure the degradation rates for both Cdc25^String^ and Cdc25^Twine^ in embryos mutant for DNA checkpoint proteins (*chk1* and *chk2*) (Figure 2b). Consistent with previous work, the degradation dynamics for Cdc25^Twine^ is similar in both wild-type and DNA replication checkpoint mutants. But whereas in wild-type embryos Cdc25^String^ is slowly degraded in the cycles leading up to the MZT by a gradual increase in degradation rate, in DNA checkpoint mutants Cdc25^String^ is targeted for degradation only at the onset of the MZT (Figure 2b). This dynamic is similar to the degradation dynamic of Cdc25^Twine^, although the increase in Cdc25^String^ degradation rate is less abrupt. Thus, in the absence of the DNA replication checkpoint, Cdc25^String^ still gets targeted for degradation, only much later, similar to Cdc25^Twine^. We note that, while we had previously shown that a Cdc25^String^-GFP protein is degraded in a DNA replication checkpoint-dependent manner [11], similar analysis argued that Cdc25^String^ is insensitive to the DNA replication checkpoint [12]. While it is possible that this difference stems from our use of transgenes lacking the endogenous 5ʹUTR and 3ʹUTR, our approach allows us to infer protein half-life regardless. Collectively, these observations argue that the differential protein stability of Cdc25^String^ and Cdc25^Twine^ is mediated by the DNA replication checkpoint and that their MZT-specific degradation might involve the new domain we have identified.

Finally, we set out to identify genomic regions responsible for targeting Cdc25^Twine^ for degradation. To this end, we generated novel lines expressing Cdc25^Twine^-GFP from a genomic construct previously shown to rescue *cdc25^twine^* mutants [9]. We crossed flies carrying these constructs to compound chromosome flies to quickly scan through the genome for loci responsible for Cdc25^Twine^ degradation [21,22]. The embryos which showed a clear delay in the degradation (Figure 2d) as well as a significant number of extra divisions (70%, N = 10) were the ones lacking the left arm of chromosome III, referred to here as 3 L^−^ (notice that the number of extra divisions were computed in embryos which do not carry any Cdc25^Twine^ transgene, as the higher Cdc25^Twine^ expression might alter the frequency of extra divisions). Thus, our observations suggest that one or more genes on the left arm of chromosome III is responsible for Cdc25^Twine^ degradation. The gene *tribbles*, which is located on chromosome 3 L, encodes a pseudo-kinase which has been shown to play an important role in targeting Cdc25^Twine^ for degradation [12]. Cdc25^Twine^ dynamics in embryos homozygous for a deficiency covering the *tribbles* locus were also significantly delayed (Figure 2d), in line with both previous findings [12] and more recent ones also quantifying Cdc25^Twine^ degradation kinetics [14]. Next, we calculated the decay time of Cdc25^Twine^ in these embryos and found that while it increases in both 3 L^−^ and *tribbles*^−^ embryos, the increase in decay time is significantly more pronounced in the 3 L^−^ embryos (Figure 2e; Supp. Figure 1). Thus, we argue that there must be at least one more gene on the left arm of chromosome III which plays an important role in the degradation of Cdc25^Twine^. This idea is in line with previous experiments showing that the number of extra divisions observed in embryos lacking the left arm of chromosome III (70%, N = 10) is significantly higher than observed in embryos mutant for *tribbles* (10%, N = 27 as reported by Liu *et al*.) [23]. One possibility is the other gene responsible for the extra divisions is *frühstart*, which is also located on chromosome 3 L, and is a known mitotic inhibitor expressed following the last nuclear cycle [24]. However, the mechanism by which Frühstart inhibits mitosis has been shown to involve inhibitory binding to the cyclin partners of Cdk1, and therefore should have no immediate effect on the degradation dynamics of Cdc25^Twine^ [25]. Hence, we propose there may be an additional, unknown, post-translational regulator Cdc25^Twine^.

To conclude, our results, along with previous ones in the literature [5,11–13,26], suggest a model for the regulation of Cdc25^Twine^ and Cdc25^String^ involving both the DNA replication checkpoint and a MZT process (Figure 2c). Our experiments also revealed a region of about 20–30 amino acids in Cdc25^Twine,^ which is required for its degradation. While we found no known protein domains within the region, we identified within it about 12 amino acids which are rather similar in Cdc25^String^ and Cdc25^Twine^. Such domain is not present in Cdc25s of other species. At the moment the functional significance of this domain remains moot. Future work will be required to elucidate whether (and how) this domain might mediate the degradation of Cdc25^Twine^ at the MZT.

## Supplementary Material

Supplemental Material

## Data Availability

The data that support the findings in this study are available upon request.
