# Supplementary figures and images for: Structure-function analysis of Cdc25Twine degradation at the Drosophila maternal-to-zygotic transition
Source: Fly (Austin). 2022 Feb 28;16(1):111–7. doi: 10.1080/19336934.2022.2043095 (PMC8890428; doi:10.1080/19336934.2022.2043095)

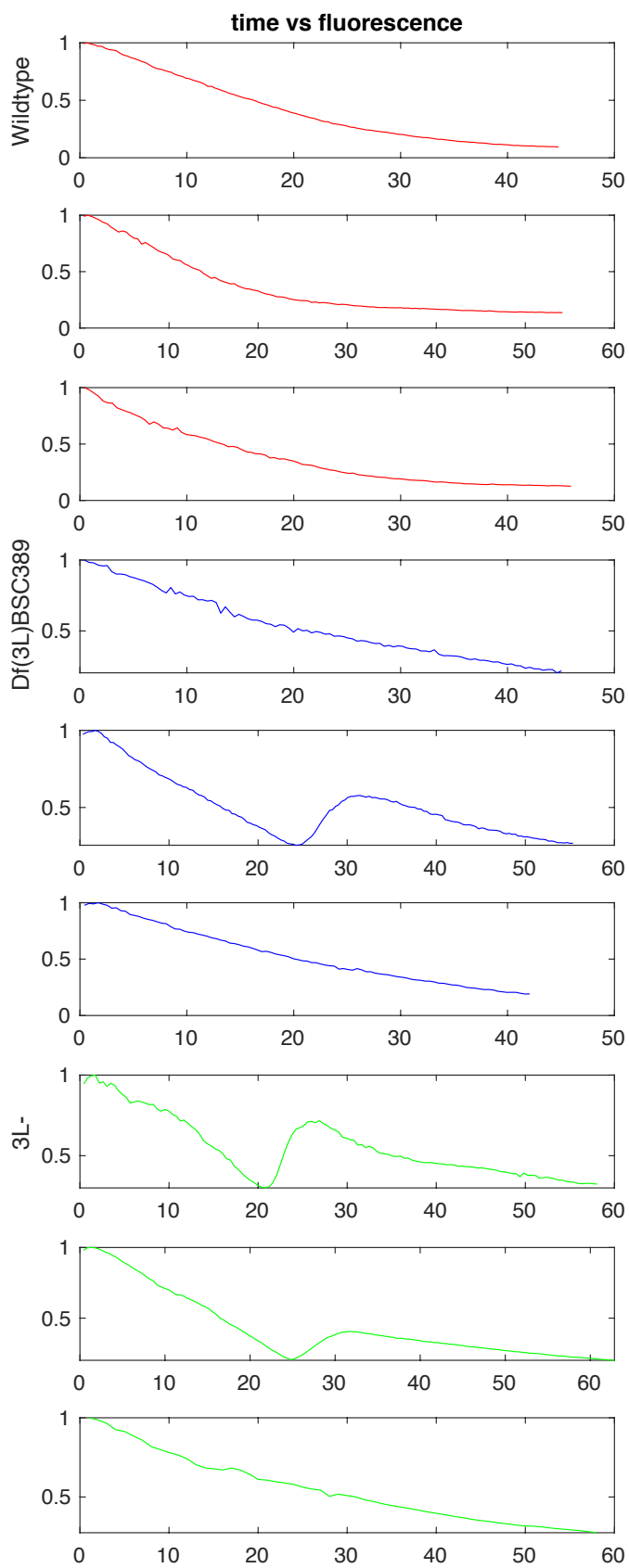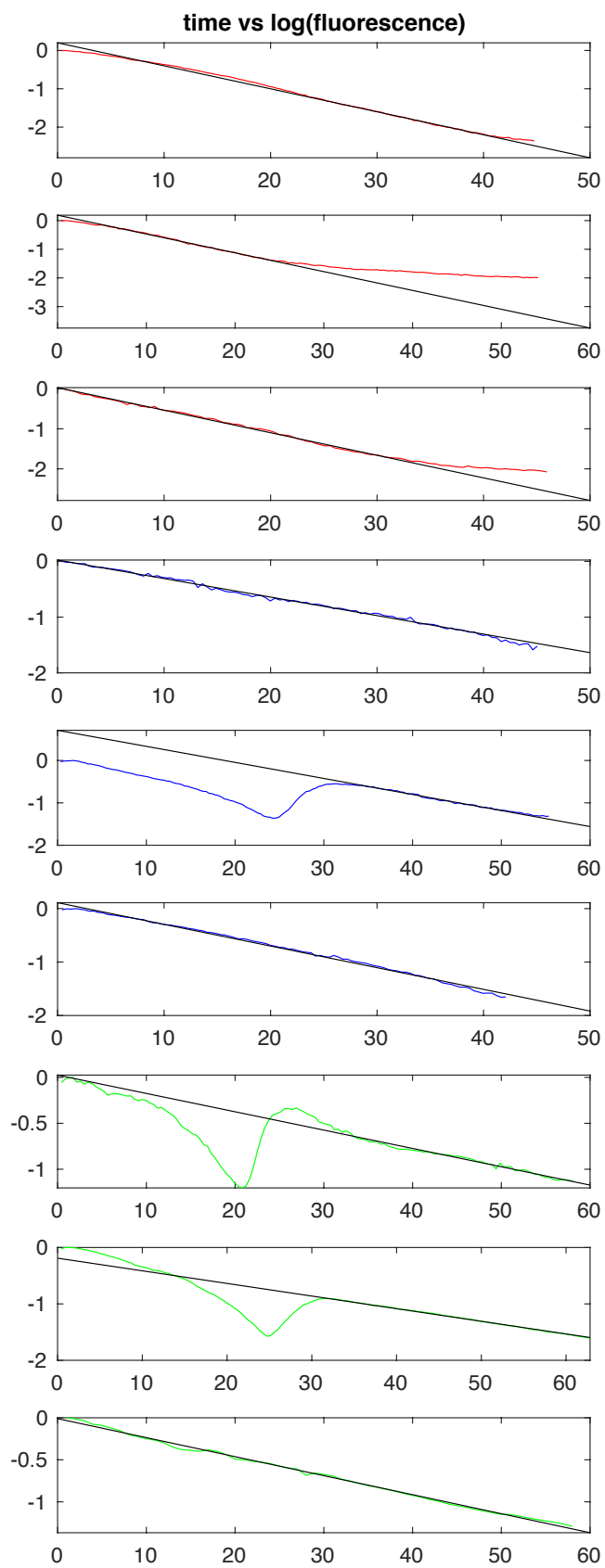

Supplement: Supplemental Material [file KFLY_A_2043095_SM4380.pdf]
